# Our Hospitals—To-Day and To-Morrow

**Published:** 1922-12

**Authors:** R. W. Harris, G. H. Hamilton


THE
### HOSPITAL and HEALTH
REVIEW

### OUR HOSPITALS—TO-DAY AND TO-MORROW.

FOR many years it has been the custom and privilege of this journal and its predecessor, "The Hospital," to place before the public at this season some account of the voluntary hospitals, collectively and individually. This has been so because we have always felt that one of the most important features of the voluntary system is the outlet it provides for the practical beneficence which is a characteristic of our countrymen, and that, at the season of Good Will, this feeling especially is extended by those who are given health and wealth towards those who are not so well endowed.

We have thought also that the many who make use of organised charity as a medium for their gifts have felt, particularly in recent years, that institutions and people who shew an inclination to help themselves are most worthy of help. If we are right in thinking that this view of giving is general there can be no doubt that the voluntary hospitals of the country have a specially strong claim at the present time. These institutions have been passing through a critical period. Faced generally, as was shewn in the leading article of our November issue, by a deficit for last year of at least £1,000,000, coming after a deficit in the previous year of a similar amount, the outlook of the system as a whole was grave in a high degree.

Unfortunately the hospitals had never seriously had the real advantage of being tried by adversity. Occasional inadequacy of funds was easily rectified by means of the appeal. To a large extent the voluntary system was the spoilt child of the benevolent. Even during the war, when so much money was required and given for other objects, the hospitals of the British Isles were prosperous. In both 1917 and 1918 there was surplus income amounting to three-quarters of a million, and in the last-named year half of this sum belonged to London. The rainy day seemed very far off and the redistribution of wealth caused by the war was not recognised until it was close upon us. Those who used to give could not continue to give as largely or, what is more in accordance with the facts, could not increase their contributions to meet the increased expenditure of the hospitals.

It must be said that as soon as the real state of affairs was clearly recognised the hospitals began at once to put their house in order, and, in finding fresh sources of income, had to adapt themselves to changed conditions. Inevitably the whole nature of the public service rendered had to be modified, and must further be modified. The hospital patient of to-day and to-morrow is not in many cases the hospital patient of yesterday. The service is being extended to a population which is not altogether in class the same as that formerly served. We believe also that the widespread opportunity to the poorer members of the community of supporting institutions, primarily intended for their own benefit—by means of the small contribution, the patient's payment, and savings associations having an insurance element—is an excellent thing in its educational aspect, bringing home to the population, in a way which has never been attempted before, the nature, aims, and needs of our splendid hospital system. Further, the new movement leaves untouched and unchanged the irresistible claim that the hospitals have and always will have upon all classes, rich and poor, in respect of the training of doctors and nurses—work which brings its influence into every home in the land.

In our opinion it will be necessary and wise for the hospitals to proceed further on American lines. In a large number of transatlantic hospitals the rich patient largely makes up the deficiency in the poor patient's payment. All classes are treated under the same roof in graduated accommodation. But in the United States the hospitals have been built and founded to provide for this system, and necessarily our hospitals would have to alter their buildings and in some cases their constitutions to provide for such a change. But in a large degree it will surely come, and the death knell of the inefficient nursing home will be sounded.

From the point of view of economy, in the sense of the word which means the proper and exact employment of material in the way in which it will do the most good, the fierce light that has beat upon hospitals during the last few years, enabling those within as well as those without to study the hospital question in all its bearings, has had an effect beyond merely increasing revenue and keeping down expenditure. It has helped to impress on us the necessity for a survey of the health services of the country, whether voluntary or rate-supported, as a whole, and to consider in what way the entire health machinery can be made to work together harmoniously without wasted effort and overlapping. As regards institutional provision for the sick, the old rule that poor-law hospitals were for chronic and hopelessly indigent people and the voluntary hospitals for curable cases amongst wage-earning classes was never in practice exactly carried out. In respect of many patients the infirmaries, generally admirable institutions, have been doing work indistinguishable from that of the voluntary hospitals.

This problem of the overlapping of the Poor Law Infirmary with the work of the Voluntary Hospitals is one only of many questions which confront the thinking citizen who is anxious to do the right thing when he is invited to come to the support of the hospitals. The expenditure on our whole Poor Law system, he learns, is enormous ; in all, it is probably well over 20 million pounds per annum. How much of this relates to institutional treatment and medical services generally probably cannot be stated with any close approximation, but its very vagueness is disturbing. The Poor Law system stands condemned as wasteful, because it overlaps, and because of its inherent defect of being administered by an authority having no responsibility for the prevention of sickness. The Government in 1918-9, when the Ministry of Health Bill was before Parliament, accepted it as a responsibility, and as a matter of urgency, that they should give effect to that part at least of the recommendations of the Maclean Committee which involved the transfer of all services relating to the sick and infirm from the Poor Law to the general Health Authorities. The " exigencies of the Parliamentary situation " have delayed this essential reform, and in the meantime the question becomes more involved by the growing practice on the part of some of the excellently-equipped Poor Law infirmaries of receiving paying patients.

Every Local Authority in this country has therefore a direct interest in the maintenance of our Voluntary Hospitals. When the transfer of the Poor Law Health Services comes, as come it must, the first concern of the Public Health Authorities must be to see that their responsibility for institutional treatment is so defined as to exclude absolutely the sphere of skilled surgical and consultative treatment. If this is not done, the Public Health Authority will otherwise, with the Poor Law " taint " removed from the Infirmaries, gradually slide into the position in which it will be saddled with the whole responsibility which is at present borne by our Voluntary Hospitals. Our hospitals have therefore the right to look to the Local Authorities for support.

We may take it for granted that if the Government are satisfied that it is a real measure of economy to abolish the Poor Law Health services and impose a fresh liability for the maintenance of the indigent sick persons on the general Public Health Authorities, this will be done, and the close and clear definition of spheres of responsibility which is so badly needed will, at the same time, be brought about. Skilled surgical and consultative treatment, it will assuredly be found, will remain essentially the province of our Voluntary Hospitals. If the thinking citizen is assured of this, he will appreciate that he is not being asked to subscribe in order to relieve the rates ; if, further, he is satisfied that the Voluntary Hospitals are developing to the utmost extent the principle of requiring that every patient shall pay up to the reasonable limit of his capacity, the generous citizen will have the real satisfaction of knowing that every pound and every shilling given is going directly to the relief of suffering in those cases only in which—and to the extent to which—the sufferer is unable to pay for such relief.

In the meantime, no sufferer must be turned away for lack of funds due to any hesitation in the minds of the thinking citizen with regard to the problems at which we have glanced. It is unquestionable that if every bed in every institution in the country, including all the Poor Law Infirmary beds, were occupied at the present time, there would still be a great deal of suffering left without adequate medical attention, and that patients would be dying at home whose lives might be saved. And, if only to show appreciation of the fact that our hospitals are helping themselves to surmount a grave crisis, we earnestly beg for a generous measure of support at the present time, all along the line.

